# Improvement of Freeze-Dried Survival of *Lactiplantibacillus plantarum* Based on Cell Membrane Regulation

**DOI:** 10.3390/microorganisms10101985

**Published:** 2022-10-07

**Authors:** Shumao Cui, Kai Hu, Zhihao Qian, Bingyong Mao, Qiuxiang Zhang, Jianxin Zhao, Xin Tang, Hao Zhang

**Affiliations:** 1 State Key Laboratory of Food Science and Technology, Jiangnan University, Wuxi 214122, China; 2 School of Food Science and Technology, Jiangnan University, Wuxi 214122, China; 3 National Engineering Research Center for Functional Food, Jiangnan University, Wuxi 214122, China

**Keywords:** *Lactiplantibacillus plantarum*, freeze-drying, cell membrane

## Abstract

The cell membrane of *Lactiplantibacillus plantarum* is a key structure for cell survival. In this study, we aimed to improve the lyophilization resistance of *L. plantarum* by regulating the cell membrane structure. Unsaturated fatty acids or cell membrane-regulating substances were added during culturing to determine their effect on the composition of cell membrane fatty acids and the survival rate of the cells after freeze-drying. The results showed that Tween 80, β-carotene and melatonin increased the lyophilization survival rate of *L. plantarum* by 9.44, 14.53, and 18.34%, respectively. After adding a lyophilization protective agent at a concentration of 21.49% at a 1:1 ratio, a combination of Tween 80, melatonin, and β-carotene was added to regulate the cell membrane, which increased the lyophilization survival rate by 32.08–86.05%. This study proposes new research directions and ideas for improving the survival rate of probiotics for industrial production.

## 1. Introduction

*Lactiplantibacillus plantarum* is a commonly used probiotic in the food industry [1]. Vacuum freeze-drying is currently the most convenient method for preparing *L. plantarum*. Screening efficient lyophilization protectors and optimizing the lyophilization process are the main means of improving the bacterial survival rate following lyophilization [2]. However, improvement of these methods has reached a bottleneck. The structural composition of cell membranes is closely related to the bacterial response to stressful environments; therefore, improving bacterial resilience by improving the structure of the cell membrane may be the key to breaking through this bottleneck [3].

Differences in cell membrane structure and components largely determine differences in cell membrane function. For example, unsaturated fatty acids make cell membranes more fluid and prevent cell death [4]. Additionally, when exogenous fatty acids are present in culture, the degree of fatty acid unsaturation in the cell membrane increases, making the membrane more fluid. Studies also show that environmental stress can lead to changes in fatty acid composition [5,6,7]. Cells also require a membrane lipid bilayer with good fluidity to maintain normal function [8]. A well-flowing membrane lipid bilayer is also an important structure for bacteria to resist a harsh external environment [9]. Some exogenously added substances, such as β-carotene [5,10,11], cyclodextrins [12], vitamin E [13], vitamin K1 [14,15], cholesterol [16], sterols [17] and melatonin [16,18], can be anchored to the membrane lipid bilayer, where they interact with the lipids to form a new lipid raft-like structure that improves the fluidity of the membrane and the viability of bacteria in harsh environments. For example, β-carotene and cyclodextrins can regulate the lipid phase of the membrane and anchor their polar groups to the opposite polar region of the membrane [19]. In addition, β-carotene interacts with van der Waals and the acyl chains of lipids, increasing the degree of freedom of the lipid heads and the fluidity of the cell membrane when it is below the phase change temperature. Similarly, vitamin E also improves membrane fluidity [20]. Melatonin can bind to membrane protein receptors on the membrane surface as a hormone [21,22] and can be used to bind to membrane protein receptors on the cell surface, thereby regulating the cell membrane. 

This study verified whether these factors potentially regulating membrane lipid bilayer structure and membrane surface matter can improve the survival rate of bacteria after lyophilization. Due to the difference in the structure of the regulatory target and mechanism, if these factors are combined, an optimal combination might be obtained. In addition, the cell membrane of *L. plantarum* N8 was observed before and after lyophilization and analyzed using scanning electron microscopy to determine whether regulation of the cell membrane could reduce the damage caused by freeze-drying. The optimum protocol was validated against other lactobacilli species, including *Lacticaseibacillus rhamnosus*, *Limosilactobacillus reuteri*, *Limosilactobacillus fermentum* and *Lacticaseibacillus casei*.

This study aimed to overcome the current bottleneck and explore a new way to improve the lyophilization stress resistance of probiotics and reverse freeze-drying-induced damage to *L. plantarum* to some extent. The findings of this study may provide insights into the alterations of cell membrane structure to produce more effective, safe, and stable probiotic freeze-dried products in the future. Further, industrialization breakthroughs and upgrades in probiotic production may soon be realized.

## 2. Materials and Methods

### 2.1. Bacterial Strains and Reagents

*Lactiplantibacillus plantarum* N8, *L. plantarum* N9, *L. plantarum* N11, *L. plantarum* CCFM8610, *L. plantarum* CCFM175, *L. plantarum* X1, *L. plantarum* CCFM242, *L. plantarum* CCFM259, *Lacticaseibacillus rhamnosus* FJND, *Limosilactobacillus reuteri* YN-DL-1-3, *Limosilactobacillus fermentum* 6-1, *Lacticaseibacillus casei* 711, all of the above bacterial strains were obtained from the Culture Collection of Food Microbiology (CCFM) at Jiangnan University (Wuxi, China).

Tween 80, Tween 20, Oleic Acid, oligoisomaltose, and glutathione were obtained from Sinopharm Chemical Reagent Co., Ltd. (Shanghai, China) β-carotene, vitamin K1, squalene, melatonin, vitamin E, cholesterol, stigmasterol, and β-cyclodextrin were purchased from Saen Chemical Technique Co., Ltd. (Shanghai, China).

### 2.2. Bacterial Culture Conditions

After scribing culture, single colonies were selected and inoculated in liquid De Man Rogosa Sharpe medium to obtain highly viable seed liquid. Tween 80-free “mMRS medium” was used for *L. plantarum* cultures, which is short for “modified MRS medium”. The bacterial strains were cultured at 37 °C.

### 2.3. Preparation of the Bacterial Suspension and Preparation before Lyophilization

The activated *L. plantarum* seed solution was added to 1 L of mMRS liquid medium at a 2% (*v*/*v*) inoculation concentration. Cultivated, and the precipitate was collected after centrifugation at 8000× *g* for 15 min. 

The bacterial sludge and lyophilization protective agent solution were mixed at a 1:1 mass ratio, shaken until fully uniform, adjusted to a pH of approximately 6.4, and placed in a 4 °C refrigerator ready for lyophilization. Lyophilization protective agent solution (100 mL) contained 21.49 g of oligoisomaltose, 7.16 g of collagen, 0.6 g of magnesium sulphate, 0.45 g of glutathione and 0.3 g of manganese sulphate. Tween 80-free mMRS medium was used in the control group to avoid any effect on the structural composition of the cell membrane. 

### 2.4. Determination of Survival Rate and Number of Live Bacteria in Lyophilization

Powder was added to saline to rehydrate it. Colonies were counted before lyophilization and after rehydration, and the survival rate was calculated according to Equation (1). The number of live bacteria per unit of bacterial powder was calculated according to Equation (2).

(1) $$\mathrm{Freeze}\text{-}\mathrm{dried}\;\mathrm{survival}\;\mathrm{rate}\;(\%)=\frac{\mathrm{Number}\;\mathrm{of}\;\mathrm{live}\;\mathrm{bacteria}\;\mathrm{after}\;\mathrm{lyophilization}}{\mathrm{Number}\;\mathrm{of}\;\mathrm{live}\;\mathrm{bacteria}\;\mathrm{before}\;\mathrm{Lyophilization}}\times 100$$

(2) $$\mathrm{Number}\;\mathrm{of}\;\mathrm{live}\;\mathrm{bacteria}\;\mathrm{per}\;\mathrm{unit}\;\mathrm{of}\;\mathrm{bacterial}\;\mathrm{powder}\;\left(\frac{\mathrm{CFU}}{g}\right)=\frac{\mathrm{Number}\;\mathrm{of}\;\mathrm{live}\;\mathrm{bacteria}\;\mathrm{after}\;\mathrm{lyophilization}}{\mathrm{Lyophilized}\;\mathrm{bacterial}\;\mathrm{powder}\;\mathrm{quality}}$$

### 2.5. Acid Stress Treatment

The seed solution was inoculated into mMRS liquid medium at a 2% (*v*/*v*) inoculation concentration and incubated for 12 h. Following this, the mixture was centrifuged at 8000× *g* for 15 min, after which the precipitate was resuspended in mMRS medium of pH 3.5 or 4.5 for 2 or 4 h.

### 2.6. Cold Stress Treatment

Seed solution was inoculated into mMRS liquid medium at a 2% (*v*/*v*) inoculation concentration, incubated for 12 h, and transferred to a 4 °C refrigerator for cold stress for 2, 4 or 6 h. The mixture was centrifuged at 8000× *g* for 15 min in order to collect the organisms prepared for subsequent lyophilization and studies. 

### 2.7. Osmotic Stress Treatment

The seed solution was inoculated into mMRS liquid medium at a 2% (*v*/*v*) inoculation concentration and incubated for 12 h. NaCl was used to adjust the osmolality of the mMRS medium to 650, 750, or 850 mOsm/kg. The mixture was centrifuged at 8000× *g* for 15 min in order to collect the organisms prepared for subsequent lyophilization and studies. 

### 2.8. Fatty Acid-Regulated Bacterial Culture

We prepared a medium by adding various fatty acids to a medium after dissolution in 1 mL absolute ethanol. The seed liquid was inoculated into the liquid medium supplemented with a fatty acid at a 2% (*v*/*v*) inoculation concentration and cultured for 12 h. The mixture was centrifuged at 8000× *g* for 15 min in order to collect the organisms prepared for subsequent lyophilization. 

### 2.9. Effects of Cell Membrane-Regulatory Substances on the Bacterial Cultures

β-carotene, vitamin K1, squalene, melatonin, vitamin E, cholesterol, sterol and β-cyclodextrin are commonly used cell membrane regulating substances in lactobacilli and can alter the structure of the cell membrane when added in culture. Mediums containing the various aforementioned cell membrane-regulating substances were prepared. The seed liquid was inoculated into a liquid mMRS medium supplemented with cell membrane-regulatory substances at a 2% (*v*/*v*) inoculation concentration and cultured at 37 °C for 12 h.

### 2.10. Determination of Fatty Acid Composition of Cell Membranes

To extract the cell membrane fatty acids, *L. plantarum* seed solution was added to the mMRS liquid medium at a 2% (*v*/*v*) inoculation concentration, and methanol solution with 1 mol/L sodium methoxide was added to the bacterial slurry obtained after centrifugation at 8000× *g* for 15 min. After the centrifugation, n-hexane solution was added for centrifugation, the supernatant liquid was aspirated and sterilized by filtration, and finally, the fatty acid content was measured. To calculate the fatty acid composition of the cell membrane, the sum of the peak areas of cell membrane fatty acids was considered to be 100%. The relative mass fraction of fatty acids was calculated according to the ratio of various fatty acid peak areas to the total peak area.

### 2.11. Scanning Electron Microscopy of the Bacteria after Lyophilization

After dehydrating and freeze-drying, gold was sprayed onto the bacterial powder, which was then placed under a scanning electron microscope to observe the shape of the bacteria after different treatments.

### 2.12. Statistical Analysis

Experimental values are taken as averages of three parallel experiments and were not repeated in later statistical analysis. Significant differences between three or more groups were identified using an ANOVA, while differences between two groups were tested using *t*-tests (SPSS 16.0). Statistical significance was set at *p* < 0.05.

## 3. Results

### 3.1. Effects of Environmental Stress Treatments on the Composition of Cell Membrane Fatty Acids and the Lyophilization Survival Rate

#### 3.1.1. Cold Stress Treatment

**Figure 1:**
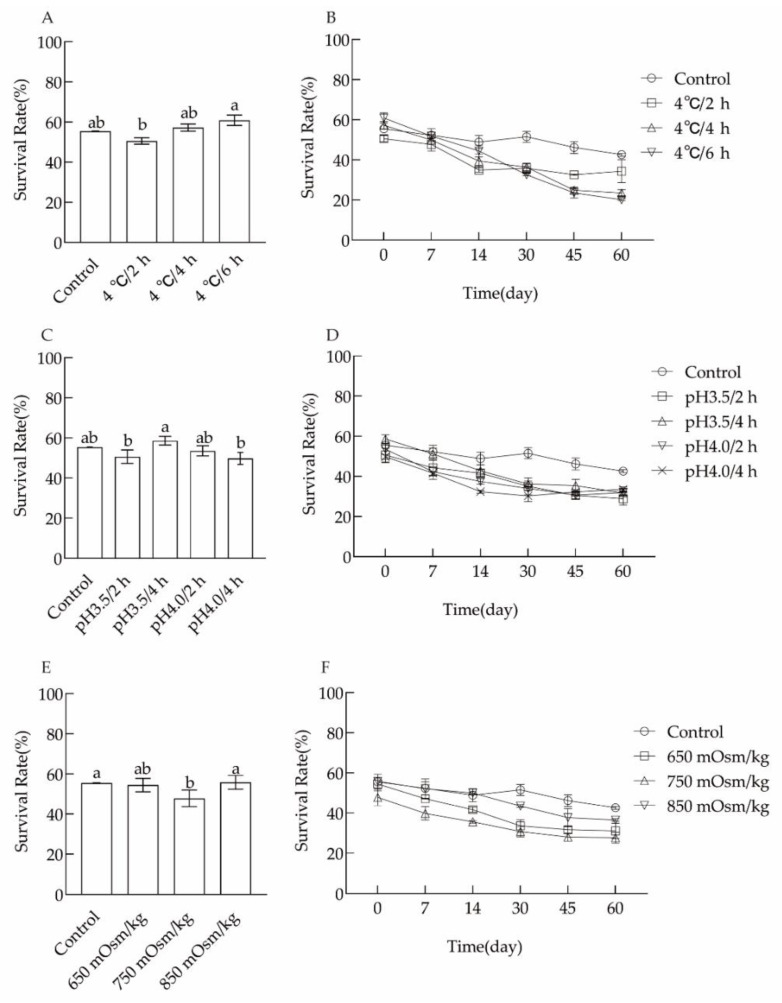
Effects of environmental stress on the lyophilization survival rate and storage of *L. plantarum*: (**A**,**B**) cold stress; (**C**,**D**) acid stress; (**E**,**F**) osmotic stress. 650, 750 and 850 mOsm/kg indicate 8 g/L, 12 g/L and 16 g/L, respectively. Different letters indicate significant differences (*p* < 0.05)

The C18:1 fatty acid of *L. plantarum* N8 was significantly increased after 6 h of treatment at 4 °C. However, cyclopropane fatty acid levels decreased (Table 1). The cell membrane fatty acid unsaturation was significantly increased, while the lyophilization survival rate of *L. plantarum* N8 did not change significantly (Figure 1A).

#### 3.1.2. Acid Stress Treatment

The changes in cell membrane fatty acids and the lyophilization survival rate of *L. plantarum* N8 after acid stress treatment are shown in Figure 1C,D and Table 2. After acid treatment, there was a significant increase in unsaturated fatty acid content in the cell membrane at pH 4.0. However, cycC19:0 decreased after treatment at pH 4.0.

#### 3.1.3. Osmotic Stress Treatment

The changes in cell membrane fatty acids and the lyophilization survival rate of *L. plantarum* N8 after osmotic stress treatment are shown in Table 3 and Figure 1E,F. When the osmolality pressure was adjusted to 650 mOsm/kg, the C18:1 and cycC19:0 contents were significantly improved, but the survival rate after lyophilization did not increase significantly.

### 3.2. Effects of Foreign Fatty Acid Addition on the Composition of Cell Membrane Fatty Acids and the Lyophilization Survival Rate

**Figure 2:**
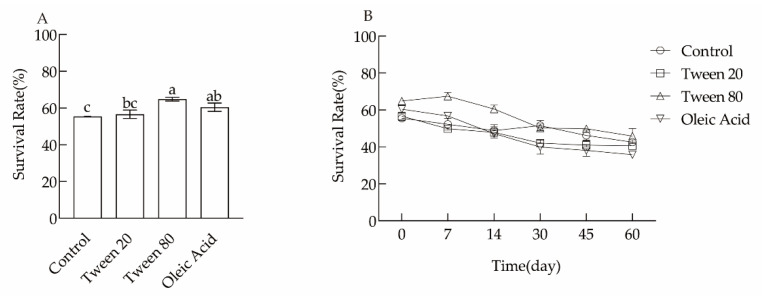
Effects of exogenous fatty acids on the lyophilization survival rate (**A**) and storage of *L. plantarum* (**B**). Different letters indicate significant differences (*p* < 0.05).

As shown in Table 4, fatty acids had a large effect on the fatty acid unsaturation of *L. plantarum* N8 cell membranes. The addition of Tween 80 and oleic acid improved the lyophilization survival rate; the change induced by adding Tween 80 was the most obvious as the fatty acid unsaturation in the cell membrane increased from 58.64 ± 0.46% to 79.15 ± 0.56%, an increase of nearly 20%. As shown in Figure 2A, the corresponding lyophilization survival rate was also significantly improved from 55.40 ± 3.45% to 64.84 ± 1.05%. As shown in Figure 2B, adding Tween 80 significantly increased the stability after storage compared to the control group. The addition of Tween 20 to the cultures was poor whose C18:1 fatty acid content in the cell membrane decreased significantly.

### 3.3. Effects of a Combination of Cell Membrane Regulatory Substances on the Lyophilization Survival Rate

**Figure 3:**
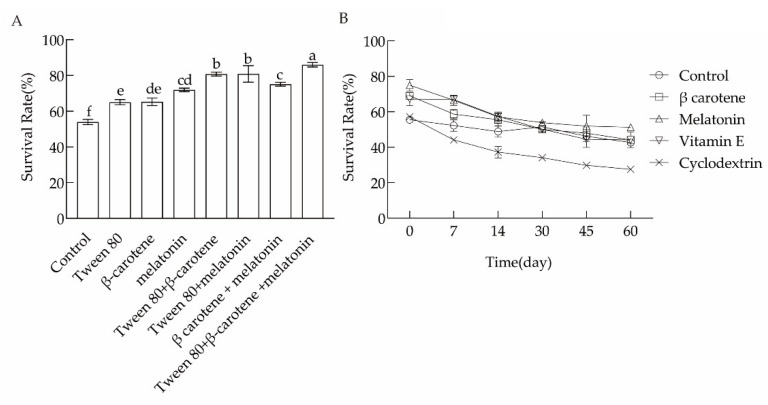
Effects of exogenous substances on the lyophilization survival rate (**A**) and storage of *L. plantarum* (**B**). Different letters indicate significant differences (*p* < 0.05).

As shown in Figure 3, β-carotene and melatonin were the most effective during storage. The storage stability of cultured *L. plantarum* N8 was significantly better than that of the control group. Melatonin, β-carotene, and Tween 80 were also used in different combinations to regulate the cell membrane structure of *L. plantarum*. The results show that the simultaneous use of three cell membrane regulatory substances improved the lyophilization survival rate of the bacteria by 32.08%, meaning an overall lyophilization survival rate of 86.05 ± 1.27% (Figure 3A).

### 3.4. Effects of Cell Membrane-Regulatory Substances on the Lyophilization Survival Rate

**Figure 4:**
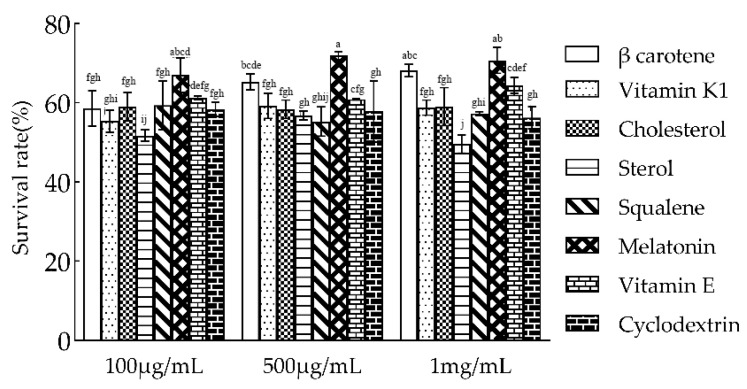
Effects of different concentrations of cell membrane regulators on the lyophilization survival rate of *L. plantarum* N8. Different letters indicate significant differences (*p* < 0.05).

As shown in Figure 4, the lyophilization survival rate varied greatly with the addition of different cell membrane-regulatory substances, which may be related to the fatty acid composition of the cell membrane of *L. plantarum* N8 and the structure of different regulators. The addition of eukaryotic cell membrane regulator cholesterol and sterol did not significantly improve the lyophilization survival rate of *L. plantarum* N8, nor did vitamin k1 and squalene. However, β-carotene significantly increased the lyophilization survival rate. This may be because the length of β-carotene is just suitable for the fatty acid composition of the cell membrane of *L. plantarum* N8. In addition, the addition of melatonin significantly improved the lyophilization survival rate. 

### 3.5. Scanning Electron Microscopy to Observe Changes before and after Cell Membrane Regulation 

**Figure 5:**
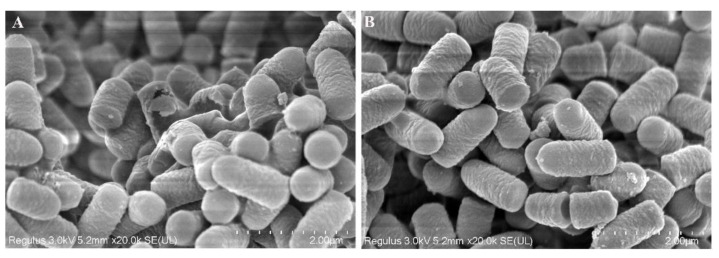
Scanning electron microscopy of lyophilized *L. plantarum* N8 powder: (**A**) ordinary mMRS culture; (**B**) Tween 80, β-carotene, and melatonin added to culture media.

As shown in Figure 5A,B, most of the cell membranes in the control group remained intact under the protection of isomaltomaltose protectors; however, some cell membranes were damaged. In addition, the cell morphology was not very turgid; the surface of some cell membranes had obvious shrinkage, and cell membrane adhesion between individual bacteria was observed, indicating that some *L. plantarum* cell membranes in the control group were damaged to a certain extent. Lyophilized *L. plantarum* powders treated with different cell membrane-regulatory substances were also observed. The cell membrane morphology was complete; no deformed cells were found, and there was no adhesion between cells. This shows that Tween 80, β-carotene, and melatonin effectively regulated the cell membranes, thus reducing damage to cell membranes during freeze-drying. 

### 3.6. The Optimal Regulation Scheme for Different Lactiplantibacillus, Lacticaseibacillus, and Limosilactobacillus Species

**Figure 6:**
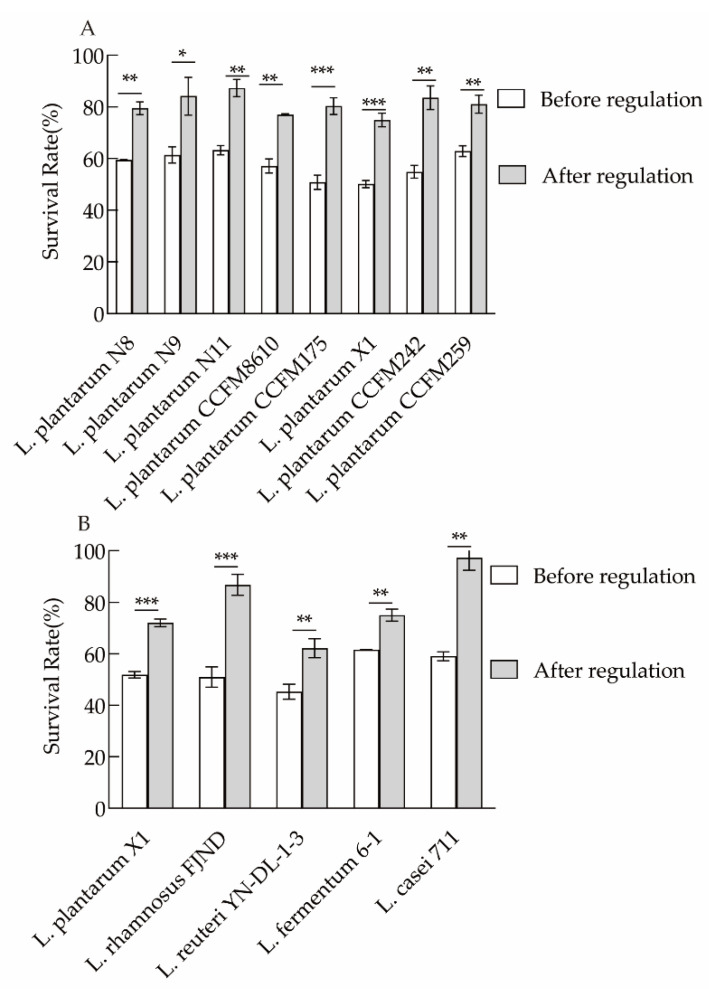
Changes in the survival rate of *L. plantarum* (**A**) and other lactobacilli species (**B**) after cell membrane regulation. Significant differences are indicated using * (*p* < 0.05), ** (*p* < 0.01) and *** (*p* < 0.001).

As shown in Figure 6A,B, the application of an optimal regulation scheme for *L. rhamnosus* FJND, *L. reuteri* YN-DL-1-3, *L. fermentum* 6-1, *L. casei* CFM711, and other strains with low lyophilization survival rates after adding Tween 80, β-carotene, and melatonin to the culture medium led to significantly improved lyophilization survival rates by up to 38.18%.

## 4. Discussion

Consistent with previous research [7], cell membranes are key structures for *L. plantarum* injury and death. We improved the lyophilization survival rate of *L. plantarum* by adjusting the structure of the cell membrane. The scanning electron microscopy results showed that the addition of certain foreign fatty acids or other substances could regulate the cell membrane; the degree of cell membrane rupture and adhesion was much smaller in the treatment group than that in the control group, thereby enhancing bacterial resistance to freeze-drying. Environmental stress treatment did not significantly improve the survival rate of the lyophilized bacteria likely because the bacteria were damaged by environmental stimuli when they were cultured at a low temperature, in the presence of an acid, or under high osmotic pressure. Cold stress can improve cellular tolerance to low temperatures during short-term freezing, but the protective effect is less pronounced after a longer storage period [23], which is consistent with the present study. The production of cold-shock proteins may be induced after cryogenic treatment to participate in cryoprotection, thereby improving bacterial lyophilization stress resistance [24]. In addition, bacteria can increase their relative content of cyclopropane fatty acids by upregulating the expression of genes and proteins related to fatty acid synthesis, which in turn enhances the fluidity of the cell membrane and protects cells from long-term growth in an acidic environment [2]. Although the cyclopropane and unsaturated fatty acid content were also increased by acid stress treatment, the lyophilization survival rate was not significantly improved. This may be related to the differences between strains and the lyophilization resistance of different strains in acidic environments. This may also be because the relative composition of unsaturated fatty acids in the cell membranes of *L. plantarum* N8 did not change significantly after acid stress. *L. acidophilus* showed an adaptive response to stress, i.e., adaptive protection [25]. This was also the case for *L. plantarum* N8, but the adaptive protection of the cell membrane after osmotic treatment was not sufficient to alleviate the damage. Therefore, hyperosmotic treatment did not improve the lyophilization survival of *L. plantarum* N8 [7]. After osmotic treatment, the resistance of *L. plantarum* N8 was reduced due to uneven pressure inside and outside the cell. The type and proportion of fatty acids in the cell membrane affect the viability of the bacteria. Wang found that adding low concentrations of oleic acid to cryoprotectant improved the lyophilization survival rate of *L. plantarum*. The effects of oleic acid and the Tween were further verified. As a result, only Tween 80 and oleic acid improved the survival rate of freeze-dried and stored *L. plantarum*. A temperature of 80 °C relative to a storage temperature of 20 °C can greatly increase the unsaturated fatty acid content of the cell membrane, thereby improving the liquidity of the cell membrane [26]. Tween 80 can induce higher cfa gene expression and better membrane fluidity during bacterial growth [27].

Subsequently, several exogenous substances that regulate the phospholipid bilayer and cell surface membrane were added to improve the stress resistance of *L. plantarum*. Research shows that sterols can act on the phospholipid bilayer of cell membranes while maintaining membrane fluidity and compressibility [28]. The poor effect of sterols in the present study may be due to the competitive relationship between sterols and cholesterol, resulting in inefficient utilization of the sterols. Melatonin acts on cell membranes and affects their fluidity [18], and different polyisoprene structures act on the surface of the cell membrane bilayer [29].

Melatonin is a class of natural hydrophilic amino acid derivatives that can promote enthalpy reduction of the non-polar chain of lipids; when melatonin is added to the lipid bilayer, it actively interacts with lipids and changes its physicochemical properties [30]. Vitamin E can also effectively stabilize bacterial morphology [31]. Studies show that the addition of vitamin E and β-cyclodextrin alters the structure of cell membranes [32], but does not significantly improve the structure of cell membrane fatty acids. In addition, the antioxidant capacity of vitamin E decreases at low temperatures; therefore, it cannot protect polyunsaturated membrane lipids from free radical attack [33]. A combination of different cell membrane-regulatory substances is more effective than one substance alone, mainly due to the anchor points at which different factors change the structure of the cell membrane [26,30]. These factors can be used to synergistically regulate cell membrane structure through different mechanisms so that bacterial viability is significantly improved compared to the addition of a single regulatory substance. The scanning electron microscopy results also support this conclusion.

## 5. Conclusions

In conclusion, regulation of the cell membrane can improve the lyophilization survival rate of *L. plantarum*. In addition, different regulatory substances were applied to various *Lactobacillus*, *Lacticaseibacillus*, and *Limosilactobacillus* species with low lyophilization survival rates, leading to significantly improved lyophilization survival. This shows that the optimal solution method to improve the lyophilization survival rate of *L. plantarum* N8, based on cell membrane regulation, applies to other species within the same genus. In this case, studying the cell membrane structure and using certain conditions to change its structure was effective. In the future, we will continue to explore the improvement of bacterial stress resistance based on the regulation of key structures related to cell survival, such as the cytoplasm or cell wall. The results of this study will aid future industrialization and commercialization of *L. plantarum* and help to develop more efficient and stable production technologies.

## Figures and Tables

**Table 1:** The composition and relative content of cell membrane fatty acids of *L. plantarum* N8 after cold stress treatment.

Methyl Fatty Acid Ester	Cell Membrane Fatty Acids Relative Mass Fraction %			
	Control	4 °C/2 h	4 °C/4 h	4 °C/6 h
C14:0	1.17 ± 0.05 a	1.23 ± 0.02 a	1.25 ± 0.01 a	1.3 ± 0.02 a
C16:0	37.92 ± 0.37 a	37.7 ± 0.2 a	35.79 ± 0.1 b	34.83 ± 0.05 c
C16:1	3.83 ± 0.08 a	3.81 ± 0.12 a	4.14 ± 0.01 a	4.18 ± 0.08 a
C18:0	2.29 ± 0.04 a	2.3 ± 0.14 a	2.05 ± 0.11 a	1.82 ± 0.15 a
C18:1	37.67 ± 0.54 c	34.35 ± 0.29 cd	38.21 ± 0.16 b	44.35 ± 0.14 a
cycC19:0	17.14 ± 0.16 c	20.63 ± 0.09 a	18.56 ± 0.12 b	13.53 ± 0.3 d
Unsaturated rate	58.64 ± 0.46 c	58.78 ± 0.08 c	60.91 ± 0.13 b	62.06 ± 0.17 a

Note: Different letters indicate significant differences (*p* < 0.05).

**Table 2:** The composition and relative content of cell membrane fatty acids of *L. plantarum* N8 after acid stress treatment.

Methyl Fatty Acid Ester	Cell Membrane Fatty Acids Relative Mass Fraction %				
	Control	pH 3.5/2 h	pH 3.5/4 h	pH 4.0/2 h	pH 4.0/4 h
C14:0	1.17 ± 0.05 a	0.92 ± 0.03 a	0.88 ± 0.03 a	1.05 ± 0.01 a	1.08 ± 0.02 a
C16:0	37.92 ± 0.37 a	36.75 ± 0.13 b	38.22 ± 0.11 a	35.28 ± 0.00 d	35.97 ± 0.90 c
C16:1	3.83 ± 0.08 ab	3.29 ± 0.04 ab	3.17 ± 0.07 b	4.12 ± 0.06 a	3.9 ± 0.01 ab
C18:0	2.29 ± 0.04 a	2.08 ± 0.03 a	2.02 ± 0.08 a	2.55 ± 0.07 a	2.17 ± 0.02 a
C18:1	37.67 ± 0.54 c	37.53 ± 0.26 c	36.03 ± 0.31 d	42.78 ± 0.10 a	41.96 ± 0.18 b
cycC19:0	17.14 ± 0.16 b	19.43 ± 0.05 a	19.68 ± 0.21 a	14.23 ± 0.10 d	14.92 ± 0.12 c
Unsaturated rate	58.64 ± 0.46 c	60.25 ± 0.10 ab	58.88 ± 0.14 c	61.13 ± 0.00 a	60.78 ± 0.08 a

Note: Different letters indicate significant differences (*p* < 0.05).

**Table 3:** The composition and relative content of cell membrane fatty acids of *L. plantarum* N8 after hypertonic stress treatment.

Methyl Fatty Acid Ester	Cell Membrane Fatty Acids Relative Mass Fraction %			
	Control	650 mOsm/kg	750 mOsm/kg	850 mOsm/kg
C14:0	1.17 ± 0.05 a	1.11 ± 0.08 a	1.03 ± 0.01 a	1.01 ± 0.02 a
C16:0	37.92 ± 0.37 a	35.58 ± 0.23 c	36.78 ± 0.08 b	37.84 ± 0.14 a
C16:1	3.83 ± 0.08 a	4.17 ± 0.05 a	4.18 ± 0.06 a	4.18 ± 0.01 a
C18:0	2.29 ± 0.04 a	2.66 ± 0.05 a	2.57 ± 0.12 a	2.53 ± 0.09 a
C18:1	37.67 ± 0.54 b	38.59 ± 0.34 a	36.83 ± 0.37 c	34.35 ± 0.14 d
cycC19:0	17.14 ± 0.16 d	17.9 ± 0.02 c	18.63 ± 0.35 b	20.09 ± 0.17 a
Unsaturated rate	58.64 ± 0.46 c	60.66 ± 0.36 a	59.64 ± 0.04 b	58.62 ± 0.10 c

Note: Different letters indicate significant differences (*p* < 0.05).

**Table 4:** The composition and relative content of cell membrane fatty acids of *L. plantarum* N8 after adding fatty acids and Tween.

Methyl Fatty Acid Ester	Cell Membrane Fatty Acids Relative Mass Fraction %			
	Control	Tween 80	Tween 20	Oleic Acid
C14:0	1.17 ± 0.05 b	2.79 ± 0.03 a	0.85 ± 0.02 b	1.28 ± 0.02 b
C16:0	37.92 ± 0.37 b	17.28 ± 0.19 d	43.36 ± 0.25 a	28.71 ± 0.31 c
C16:1	3.83 ± 0.08 a	3.49 ± 0.03 a	2.67 ± 0.07 b	2.51 ± 0.05 b
C18:0	2.29 ± 0.04 a	0.78 ± 0.02 b	1.73 ± 0.04 a	2.14 ± 0.07 a
C18:1	37.67 ± 0.54 c	50.14 ± 0.25 a	27.75 ± 0.16 d	39.26 ± 0.41 b
cycC19:0	17.14 ± 0.16 d	25.52 ± 0.23 a	23.64 ± 0.31 b	20.10 ± 0.14 c
Unsaturated rate	58.64 ± 0.46 c	79.15 ± 0.56 a	54.06 ± 0.37 d	67.87 ± 0.19 b

Note: Different letters indicate significant differences (*p* < 0.05).

## Data Availability

Not applicable.
